# The Ameliorative Effect of Silicon on Maize Plants Grown in Mg-Deficient Conditions

**DOI:** 10.3390/ijms20040969

**Published:** 2019-02-22

**Authors:** Seyed Abdollah Hosseini, Sara Naseri Rad, Nusrat Ali, Jean-Claude Yvin

**Affiliations:** 1 Plant Nutrition Department, Centre Mondial de I’lnnovation Roullier, 35400 Saint Malo, France; Nusrat.Ali@roullier.com (N.A.); JeanClaude.Yvin@roullier.com (J.-C.Y.); 2 Molecular Plant Nutrition Group, Physiology and Cell Biology, Leibniz-Institute of Plant Genetics and Crop Plant Research, 06466 Gatersleben, Germany; naseri@ipk-gatersleben.de

**Keywords:** mineral deficiency, assimilate partitioning, primary metabolite, hormonal regulation

## Abstract

The importance of magnesium (Mg) for plant growth is well-documented. Silicon (Si)-mediated alleviation of mineral deficiencies has been also reported in a number of plant species; however, there is no report on the relevance of Si nutrition in plants grown in Mg-deficient condition. Therefore, in the present work, an attempt was undertaken to study the role of Si nutrition in maize plants exposed to Mg deficiency. Plants were grown either under low (0.02 mM) or normal (0.5 mM) levels of Mg, with or without Si supplement. We have shown that Mg-deficient plants treated with Si maintained their growth and increased significantly the levels of chlorophyll and soluble sugars compared to those plants which did not receive Si. In addition, the concentrations of hexose-P, and glycolytic intermediate metabolites—mainly organic acids (isocitric and glutamic acids)—were increased in response to Si nutrition, which was associated with an increase in the levels of stress amino acids such as gamma-aminobutyric-acid (GABA), serine and glycine, as well as polyamines putrescine, which overall contributed to Mg stress tolerance. In addition, Si enhanced the levels of phytohormones cytokinin *iso*-pentenyladenine (IP), *iso*-pentenyladenine riboside (IPR), jasmonic acid (JA) and its derivate l-isoleucine (JA-ILE). The increase in cytokinin maintained the growth of Mg-deficient plants, while JA and JA-IEA were induced in response to carbohydrates accumulation. Altogether, our study reveals the vital role of Si under Mg deficiency by regulating plant primary metabolite and hormonal changes.

## 1. Introduction

To ensure proper growth and development, plants require nutrients. Restricting nutrient supply will lead to poor establishment, which will subsequently arrest proper growth [1]. Among essential macronutrients, magnesium (Mg) is considered as an indispensable mineral for crop plants. The function of Mg in plants is mainly related to its capacity to interact with strongly nucleophilic ligands [2]. A major well-known function of Mg in plants is its involvement as a central atom of the chlorophyll molecule; Mg interacts into activation of porphyrin structure. Besides this crucial function, Mg plays various roles in plants, such as carbohydrate partitioning, enzyme activation, stabilizing specific conformations of nucleic acids required for their synthesis and function, as well as protein synthesis [2,3].

Mg concentrations in soil solutions are usually between 125 and 8.5 mM; however, plants, which are typically immobile encounter a lack of Mg in their natural environment [2]. Most of the Mg is incorporated as structural mineral, and therefore, is not readily available for plants. In addition, the soil properties like pH and type of clay also have a direct effect on Mg uptake and availability for plants [3]. Mg deficiency diminishes plant growth, resulting in necrosis spots on shoot surface, shorter root, and smaller leaves. The Mg deficiency symptoms typically appear on mature leaves due to transport of Mg from old to young leaves, which is eventually increased by the breakdown of structural proteins of the thylakoids. That is why the plastid pigments are affected similarly to chlorophyll molecules in Mg-deficient plants. Another consequence of Mg deficiency in plants is the impairment of carbohydrate partitioning, which can be even detected well in advance of visibility of Mg deficiency symptoms. Carbohydrate partitioning is disturbed by impaired phloem loading of sugars, which develops into an accumulation of carbohydrates in leaves, resulting in lower carbohydrate export to the sink organ, which strongly reduces root growth [2].

Silicon (Si) is the second most abundant element in the earth’s crust, and is considered a beneficial element for agriculture production [4]. The supply of Si has been shown to mitigate the adverse effect of numerous abiotic stresses, including drought, salt and heavy metals [5,6]. More recently, efforts into understanding how Si alleviates mineral nutrient deficiencies like potassium [7,8], sulfur [9], nitrogen [10] and phosphorus [11,12,13] have led to researchers focusing on the role of Si nutrition in crop plants. Moreover, the role of Si on plant–pathogen interactions and stimulating plant defense responses against pathogen attack has been well described [14,15]. However, there are no case studies demonstrating the cross talk between Si and Mg nutrition in plants.

Hence, the aim of this study was to determine whether, and to what extent, Si application mitigates Mg deficiency in maize plants. We hypothesized that Mg deficiency would induce a decline in plant chlorophyll levels and impair phloem loading of sugar to the roots, which would result in photosynthesis inhibition and accumulation of sugar in the leaves, thus reducing plant growth. The application of Si to Mg-deficient plants increases chlorophyll level and biomass, enhances sugar accumulation and modulates hormonal changes, thus alleviating Mg deficiency. To accomplish the objective of this study and to investigate the cross-talk between Si and Mg, different physiological, biochemical and molecular approaches were performed.

## 2. Results

### 2.1. Influence of Si Supply on Plant Growth and Chlorophyll Levels under Mg Deficiency

**Figure 1:**
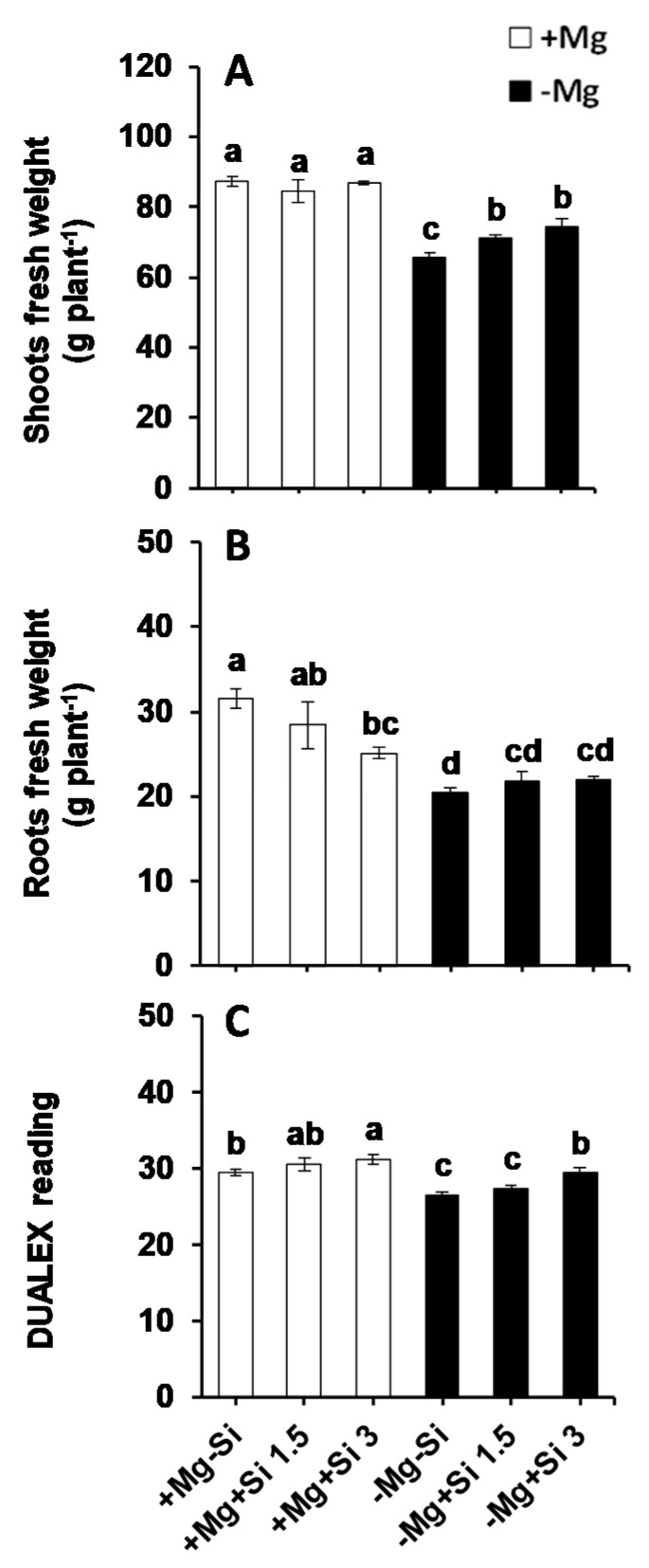
Influence of Si supply on roots and shoots biomass as well as chlorophyll level of maize plants subjected to Mg deficiency. (**A**) shoot fresh weights; (**B**) root fresh weights and (**C**) chlorophyll level measured by dualex device. Plants were grown in hydroponic culture under low Mg (0.02 mM) or normal Mg (0.5 mM) supply and two concentrations of Si (1.5 and 3 mM). Si provided in the second week of plant growth in hydroponic culture when Mg deficiency was applied. 21-days old plants were harvested 14 days after imposition of Mg deficiency. The white and black bars represent Mg-sufficient and Mg-deficient plants, respectively. Bars indicate means ± SE. Different letters denote significant differences according to LSD test (*p* < 0.05; *n* = 4).

In the present study, Si supply did not influence the shoot fresh weights at normal levels of Mg (0.5 mM), whereas both concentrations of Si significantly increased shoot fresh weights under Mg- deficient conditions compared to Mg-deficient plants which did not receive Si (Figure 1A). Conversely, the root fresh weights were significantly reduced under normal levels of Mg when the concentration of Si increased in the nutrient solution (Figure 1B). Under Mg deficiency, root fresh weights were significantly decreased compared to Mg-sufficient plants, and both Si concentrations slightly but not significantly increased the root fresh weights compared to non-Si treated roots (Figure 1B). As a major determinant for Mg deficiency, we determined chlorophyll levels by a non-destructible method using a Dualex device. Compared to Mg-sufficient plants, the chlorophyll levels decreased significantly under Mg deficiency. However, Si significantly increased and maintained its level in the Mg-deficient plants which were supplied with silicon, and the levels were comparable and similar to those of Mg- sufficient plants (Figure 1C). These results indicated that Si maintained the growth of maize plants exposed to Mg deficiency by increasing the shoot fresh weight and chlorophyll levels.

### 2.2. Influence of Si Supply on Magnesium and Silicon Concentrations under Mg Deficiency

**Figure 2:**
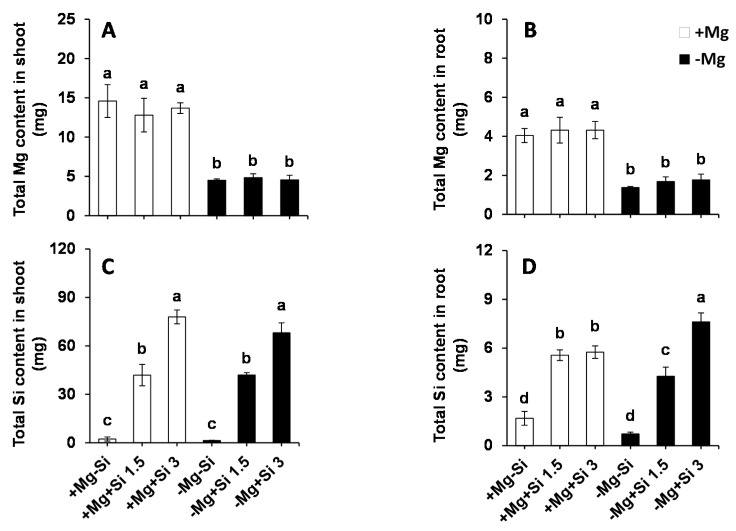
Influence of Si supply on total magnesium (Mg) and silicon (Si) content of maize plants subjected to Mg deficiency. (**A**) total Mg content in roots; (**B**) total Mg content in shoots; (**C**) total Si content in roots and (**D**) total Si content in shoots of maize. Plants were grown in hydroponic culture under low Mg (0.02 mM) or normal Mg (0.5 mM) supply and two concentrations of Si (1.5 and 3 mM). Si provided in the second week of plant growth in hydroponic culture when Mg deficiency was applied. 21-days old plants were harvested 14 days after imposition of Mg deficiency. The white and black bars represent Mg-sufficient and Mg-deficient plants, respectively. Bars indicate means ± SE. Different letters denote significant differences according to LSD test (*p* < 0.05; *n* = 4).

In our study, we examined whether Si affected Mg uptake in the roots and its translocation to the shoots. As we expected, the total Mg in Mg-deficient plants as significantly decreased compared to Mg-sufficient plants for both roots and shoots (Figure 2A,B). Apparently, Si supply did not influence the uptake and/or translocation of Mg, irrespective of the level of Mg in the solution. We then calculated the total Si content in both roots and shoots, which was found to be similar in non-Si treated plants, irrespective of the Mg levels in the nutrient solution (Figure 2C,D). Conversely, the total Si content increased significantly in a dose-dependent manner in both the roots and shoots of Si treated plants at both Mg levels (Figure 2C,D). Under Mg deficiency, the root Si content was significantly higher when the concentration of Si was increased in the medium compared to low levels of Si- and also Mg-sufficient plants (Figure 2C). Two-way ANOVA demonstrated significant interaction effects between Mg and Si in the total root Si (Pr(>F) = 0.0006) (Table S1).

### 2.3. Influence of Si Supply on Metabolite Changes under Mg Deficiency

**Figure 3:**
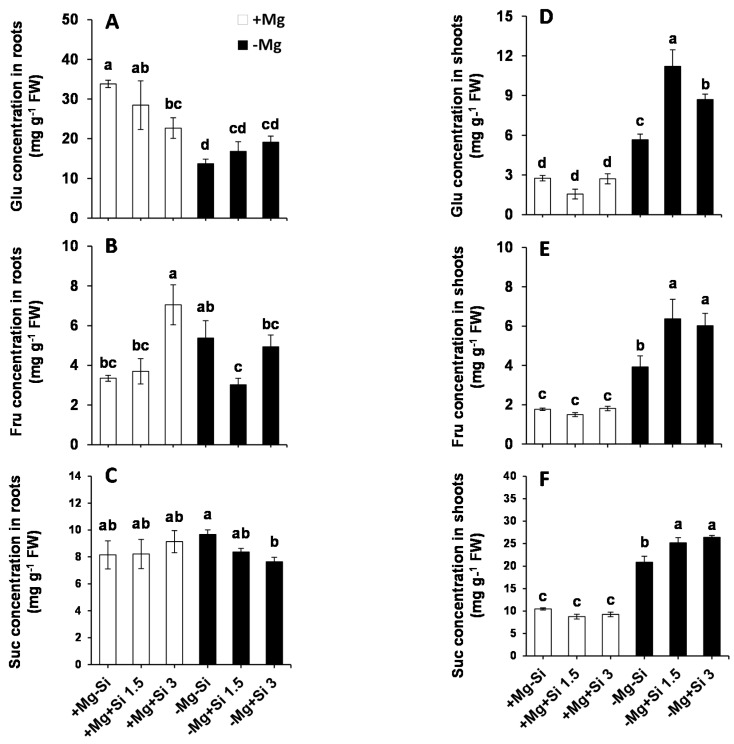
Influence of Si supply on soluble sugars concentrations of maize plants subjected to Mg deficiency. (**A**) Glu concentration in roots, (**B**) Fru concentration in roots, (**C**) Suc concentration in roots, (**D**) Glu concentration in shoots, (**E**) Fru concentration in shoots, (**F**) Suc concentration in shoots of maize. Plants were grown in hydroponic culture under low Mg (0.02 mM) or normal Mg (0.5 mM) supply and two concentrations of Si (1.5 and 3 mM). Si provided in the second week of plant growth in hydroponic culture when Mg deficiency was applied. 21-days old plants were harvested 14 days after imposition of Mg deficiency. The white and black bars represent Mg-sufficient and Mg-deficient plants, respectively. Bars indicate means ± SE. Different letters denote significant differences according to LSD test (*p* < 0.05; *n* = 4).

Due to the important role of Mg in assimilation partitioning between the roots and shoots, the level of soluble sugars were determined in the present study. In the roots of Mg-deficient plants, the level of Glucose (Glu) significantly decreased compared to Mg sufficient-plants (Figure 3A). The lower concentration of Si in the nutrient solution did not change the levels of root Glu in Mg- sufficient plants, while this level was significantly reduced with higher concentrations of Si compared to non-Si-treated Si plants (Figure 3A). We also observed a slight but not significant increase in the level of root Glu by Si supply in Mg-deficient condition (Figure 3A). However, a significant interaction between Mg and Si was found for Glu (Pr(>F) = 0.0307) (Table S1). Furthermore, the concentration of fructose (Fru) in the roots was significantly increased with higher levels of Si in Mg-sufficient plants, but this decreased under Mg deficiency with the same dose of Si (Figure 3B). Additionally, higher concentrations of Si restored the level of Fru to the same level of Mg-deficient plants (Figure 3B). The levels of root sucrose (Suc) were almost identical in Mg-sufficient plants in spite of Si application (Figure 3C), whereas under Mg deficiency, the level of roots Suc significantly decreased in a dose-dependent manner compared to Mg-deficient plants which did not receive Si (Figure 3C). It is worthy of note that although the level of Suc declined in Mg-deficient plants treated with Si, its level was similar to those in the Mg-sufficient plants (Figure 3C), which implies that the phloem loading of sugars from shoots to roots may not be disturbed. To support this finding, we measured the expression of the genes involved in Suc and/or sugar transporters in roots. Interestingly, the expression levels of sucrose transporter 1 (*ZmSUT1)* and bidirectional sugar transporters (*ZmSWEET13a*, *b* and *c)* were induced by Si in Mg-deficient plants (Figure S1).

In general, the levels of Glu, Fru, and Suc were significantly increased in the shoots of Mg-deficient plants compared to Mg-sufficient plants (Figure 3D–F). Interestingly, in Mg-deficient plants which were treated with Si, the concentrations of these sugars significantly increased compared to non-Si treated plants (Figure 3D–F). Two way ANOVA analysis also demonstrated significant interaction between Mg and Si for both Fru and Suc in shoots (Pr(>F) = 0.0007 and 0.0011) (Table S1).

**Figure 4:**
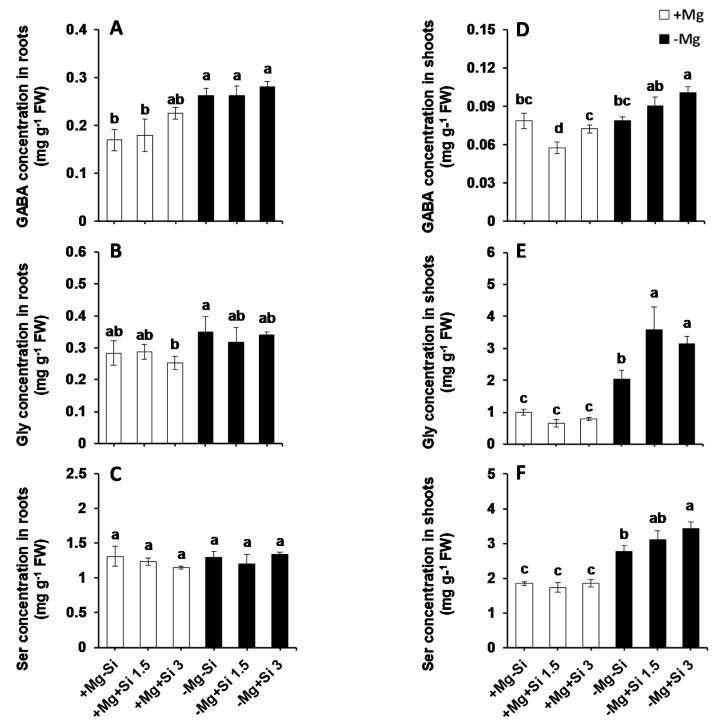
The influence of Si supply on amino acids concentrations of maize plants subjected to Mg deficiency. (**A**) GABA concentration in roots, (**B**) Gly concentration in roots, (**C**) Ser concentration in roots, (**D**) GABA concentration in shoots, (**E**) Gly concentration in shoots and (**F**) Ser concentration in shoots of maize. Plants were grown in hydroponic culture under low Mg (0.02 mM) or normal Mg (0.5 mM) supply and two concentrations of Si (1.5 and 3 mM). Si provided in the second week of plant growth in the hydroponic culture when Mg deficiency was applied. 21-days old plants were harvested 14 days after imposition of Mg deficiency. The white and black bars represent Mg-sufficient and Mg-deficient plants, respectively. Bars indicate means ± SE. Different letters denote significant differences according to LSD test (*p* < 0.05; *n* = 4).

We further analyzed the levels of amino acids, organic acids and polyamines which are thought to change under mineral nutrition deficiencies [7]. The level of most amino acids did not change by Si supply, at least under our experimental conditions (Tables S2 and S3). However, Si significantly increased the levels of important stress amino acids like gamma-aminobutyric-acid (GABA), glycine (Gly) and serine (Ser), particularly in shoots and under Mg-deficient conditions (Figure 4D–F). In the roots, the level of GABA was not changed in Mg- sufficient plants with or without the application of Si (Figure 4A). The root GABA was significantly higher in Mg-deficient plants compared to normal Mg with or without Si application (Figure 4A). There were no consistent changes in the levels of root Gly and Ser in all tested conditions (Figure 4B,C). Remarkable changes in the levels of GABA, Gly, and Ser were observed in the shoots of Mg-deficient plants which were supplied with Si (Figure 4D–F). It is worth noting that Two-way ANOVA revealed that there was also a significant interaction effect between Mg and Si in the GABA (Pr(>F) = 0.0076), and for the Gly levels (Pr(>F) = 0.0160) in shoots (Table S1).

Under normal Mg supply, the level of shoot GABA decreased with a lower level of Si, but was restored to the levels of Mg-sufficient plants with the higher concentration of Si (Figure 4D). Unlike Mg-sufficient plants, the level of shoot GABA increased with increasing Si concentration in the solution, and it was even statistically significant with the higher level of Si (Figure 4D). Si did not impact the levels of shoot Gly and Ser when plants were supplied with normal Mg; however, under Mg deficiency, Si significantly increased the level of these two amino acids in comparison with Mg-deficient plants which did not receive Si (Figure 4E,F).

**Figure 5:**
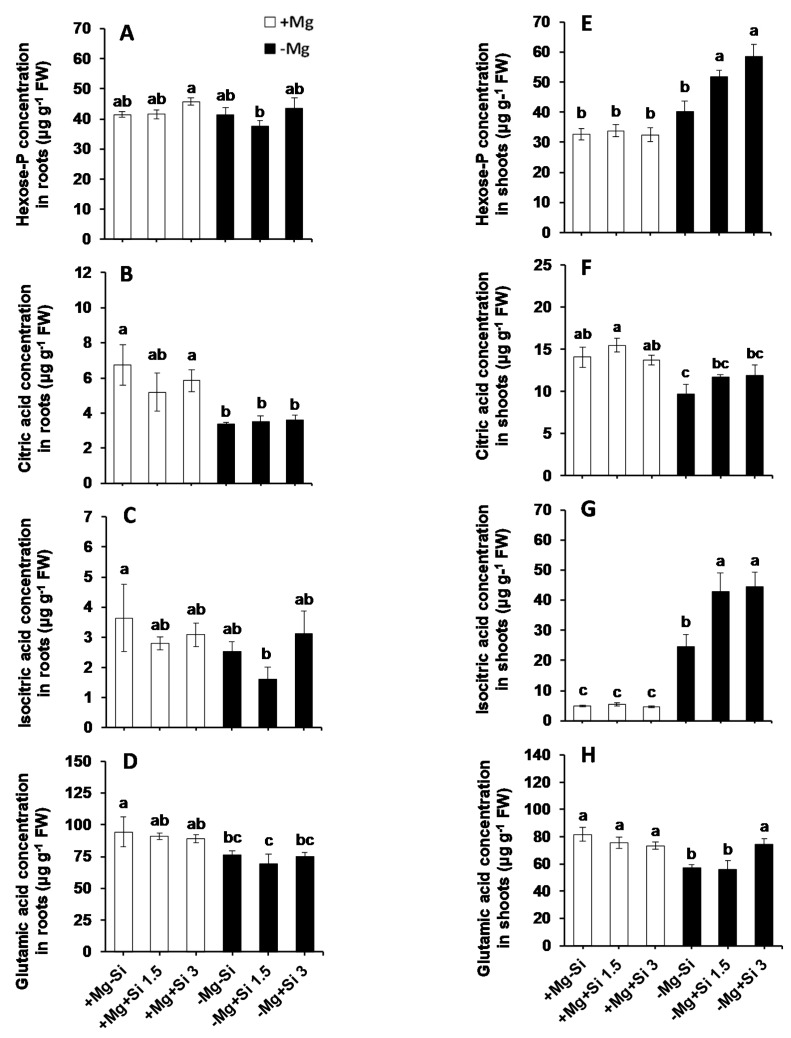
The influence of Si supply on Hexose-P and organic acids concentrations of maize plants subjected to Mg deficiency. (**A**) Hexose-P concentration in roots, (**B**) citric acid concentration in roots, (**C**) isocitric acid concentration in roots, (**D**) glutamic acid concentration in roots, (**E**) hexose-P concentration in shoots, (**F**) citric acid concentration in shoots, (**G**) isocitric acid concentration in shoots and (**H**) glutamic acid concentration in shoots of maize. Plants were grown in hydroponic culture under low Mg (0.02 mM) or normal Mg (0.5 mM) supply and two concentrations of Si (1.5 and 3 mM). Si provided in the second week of plant growth in the hydroponic culture when Mg deficiency was applied. 21-days old plants were harvested 14 days after imposition of Mg deficiency. The white and black bars represent Mg-sufficient and Mg-deficient plants, respectively. Bars indicate means ± SE. Different letters denote significant differences according to LSD test (*p* < 0.05; *n* = 4).

We further measured glycolysis intermediate metabolites including organic acids which are involved in tricarboxylic acid cycle (TCA) cycle and amino acids synthesis. In the roots, we did not observe any particular changes in the levels of hexose-P and isocitric acid (Figure 5A,C). Compared to normal Mg plants, the level of citric acid and glutamic acid were decreased in Mg-deficient plants, irrespective of the level of Si in the solution (Figure 5B,D). Like Glu and Fru, the levels of hexose-P was higher in shoots of Mg-deficient plants compared to Mg-sufficient plants, and this increase was statistically significant when Si was added to Mg-deficient plants (Figure 5E). Two-way ANOVA analysis showed that both Mg and Si, as well as their interactions, were significant for this parameter (Table S1). Consequently, the levels of organic acids like isocitric acid were significantly increased in the shoots of Mg-deficient plants compared to Mg-sufficient plants, and this increase was more pronounced when Si was supplemented (Figure 5G and Table S1). Under Mg deficiency, the level of glutamic acid in the shoots decreased in comparison to that in Mg-sufficient plants; however, a higher concentration of Si significantly restored it to the level of Mg-sufficient plants (Figure 5H).

**Figure 6:**
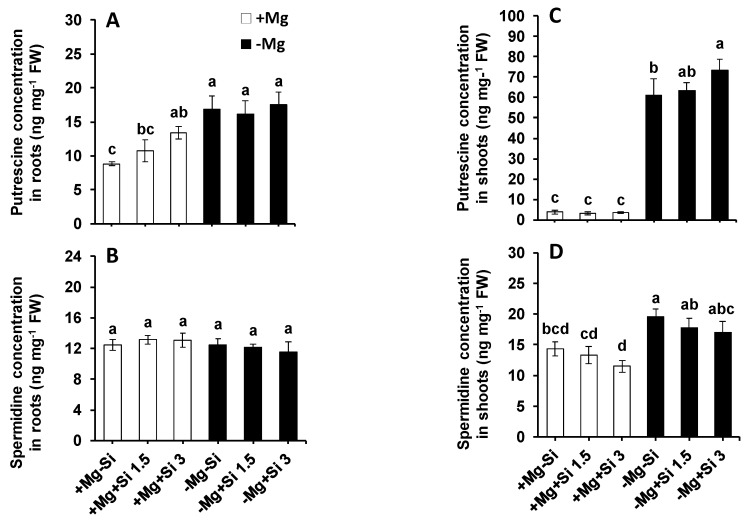
The influence of Si supply on polyamines concentrations of maize plants subjected to Mg deficiency. (**A**) putrescine concentration in roots, (**B**) spermidine concentration in roots, (**C**) putrescine concentration in shoots and (**D**) spermidine concentration in shoots of maize. Plants were grown in hydroponic culture under low Mg (0.02 mM) or normal Mg (0.5 mM) supply and two concentrations of Si (1.5 and 3 mM). Si provided in the second week of plant growth in the hydroponic culture when Mg deficiency was applied. 21-days old plants were harvested 14 days after imposition of Mg deficiency. The white and black bars represent Mg-sufficient and Mg-deficient plants, respectively. The white and black bars are belonged to Mg sufficient and Mg deficient plants, respectively. Bars indicate means ± SE. Different letters denote significant differences according to LSD test (*p* < 0.05; *n* = 4).

Among the polyamines, putrescine (Put) and spermidine (Spm) were detected using HPLC device. In roots of Mg-sufficient plants, the level of Put was increased with Si supply, and this increase was statistically significant when the concentration of Si increased in the solution (Figure 6A). The roots of Mg-deficient plants showed significantly higher Put levels compared to Mg-sufficient plants, with or without of Si supply (Figure 6A). There were no changes in the level of roots Spm in any of the tested conditions (Figure 6B). However, in the shoots, the level of both Put and Spm were significantly increased in Mg-deficient plants compared to Mg-sufficient plants (Figure 6C,D). The increase of Put was more pronounced, and even statistically higher, in Mg-deficient plants which received a higher concentration of Si (Figure 6C). The level of shoot Spm also increased significantly in Mg-deficient plants compared to Mg-sufficient plants, irrespective of Si application (Figure 6D).

### 2.4. Influence of Si Supply on Hormone Regulation Under Mg Deficiency

**Figure 7:**
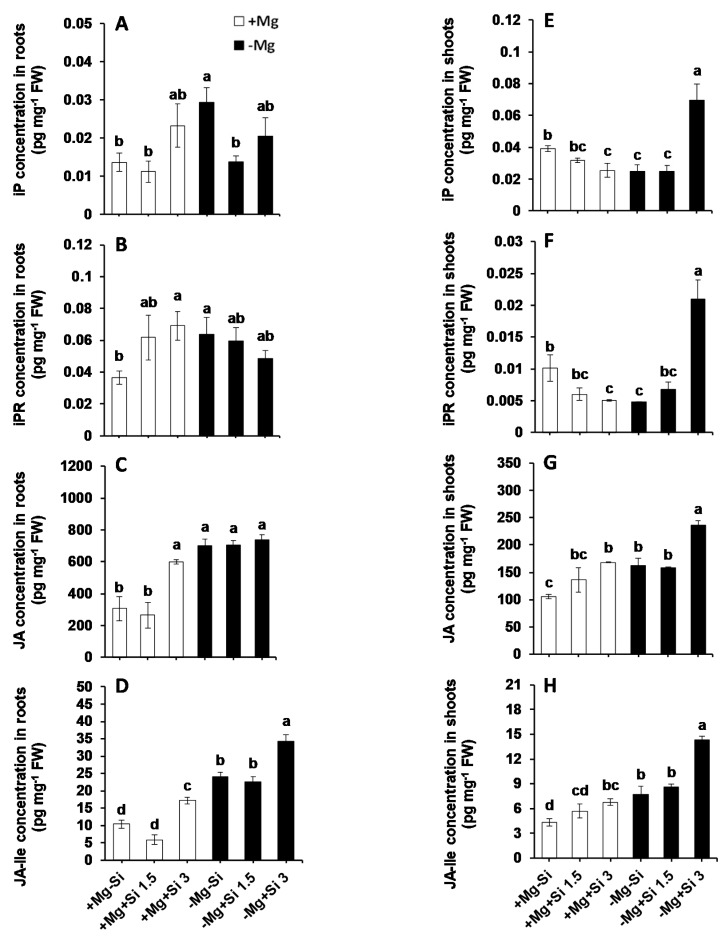
The influence of Si supply on phytohormone concentrations of maize plants subjected to Mg deficiency. (**A**) iP concentration in roots, (**B**) iPR concentration in roots, (**C**) JA concentration in roots, (**D**) JA-Ile concentration in roots, (**E**) iP concentration in shoots, (**F**) iPR concentration in shoots, (**G**) JA concentration in shoots and (**H**) JA-Ile concentration in shoots of maize. Plants were grown in hydroponic culture under low Mg (0.02 mM) or normal Mg (0.5 mM) supply and two concentrations of Si (1.5 and 3 mM). Si provided in the second week of plant growth in the hydroponic culture when Mg deficiency was applied. 21-days old plants were harvested 14 days after imposition of Mg deficiency. The white and black bars represent Mg-sufficient and Mg-deficient plants, respectively. Bars indicate means ± SE. Different letters denote significant differences according to LSD test (*p* < 0.05; *n* = 4).

To investigate the role of Si on hormone regulation under Mg deficiency, we measured the concentrations of major phytohormones in this study. The main changes were associated with the levels of active cytokinin *iso*-pentenyladenine (IP), *iso*-pentenyladenine riboside (IPR), jasmonic acid (JA), and its derivate L-isoleucine (JA-ILE). The roots of Mg-deficient plants showed significantly higher levels of IP and IPR compared to Mg-sufficient plants (Figure 7A,B). A higher concentration of Si further increased the levels of IP in a dose-dependent manner, irrespective of the Mg levels in the solution (Figure 7A). This holds true for IPR levels, but only under normal Mg levels, because IPR levels decreased by Si in Mg-deficient plants in a dose-dependent manner (Figure 7B). The levels of JA in roots of Mg-deficient plants were higher than those in the Mg-sufficient plants, without any positive effect of Si. However, under normal Mg supply, JA levels increased only when the plants were treated with a higher concentration of Si (Figure 7C). The same trend was also observed in the level of root JA-ILE, but higher concentrations of Si could increase the level of this hormone in both Mg- deficient and Mg-sufficient plants. (Figure 7D). We also observed a significant interaction effect between Mg and Si for IP (Pr(>F) = 0.0352), IPR (Pr(>F) = 0.0277), and JA-ILE (Pr(>F) = 0.0007) in the roots (Table S1).

In the shoots of Mg-sufficient plants, the levels of both IP and IPR decreased by increasing the concentration of Si, but the concentrations of both compounds increased in Mg-deficient plants which received a higher concentration of Si (Figure 7E,F). Unlike shoots IP and IPR, the levels of shoot JA and JA-ILE were increased in Mg-sufficiency by Si supply in a dose-dependent manner (Figure 7G,H). Interestingly, the higher concentration of Si significantly increased the levels of shoot JA and JA-ILE at least two times under Mg deficiency (Figure 7G,H). As for the roots, a significant interaction between Mg and Si was also observed for IP (Pr(>F) = 0.00001), IPR (Pr(>F) = 0.00004) and JA-ILE (Pr(>F) = 0.0110) (Table S1). It is worth noting that we measured the levels of all other phytohormones such as abscisic acid (ABA), 1-aminocyclopropane-1-carboxylic acid (ACC), salicylic acid (SA) and gibberellin acid (GA), but did not observe any particular changes in their levels (Table S4).

### 2.5. Influence of Si Supply on Transcriptional Regulation of the DELLA Proteins and JA Biosynthetic Pathway

**Figure 8:**
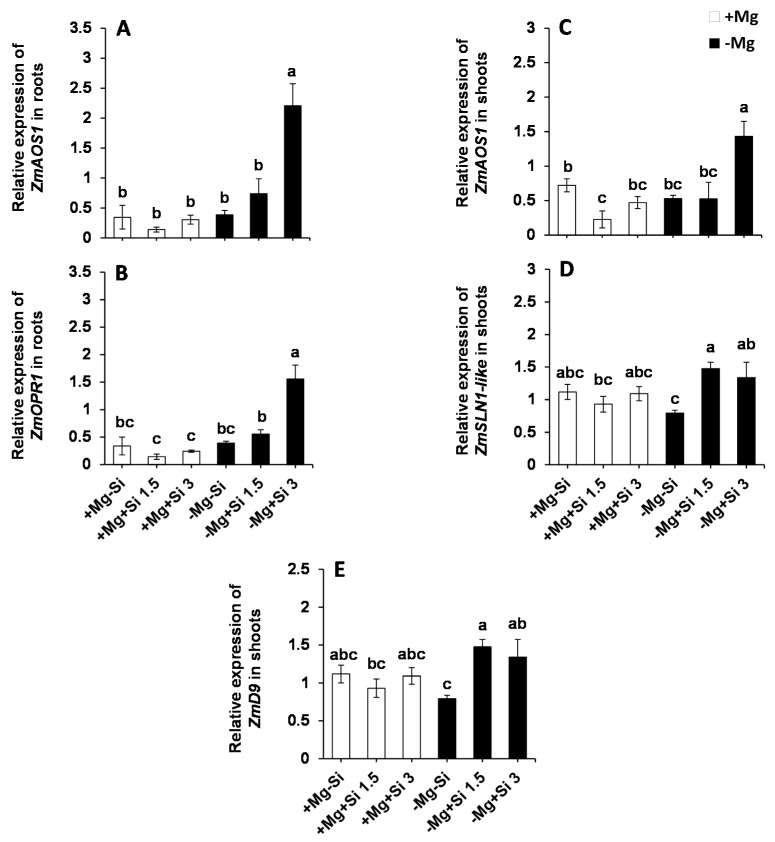
The influence of Si supply on transcriptional regulation of DELLAs proteins and JA biosynthetic pathway in maize plants subjected to Mg deficiency. (**A**) Relative expression of *ZmAOS1* in roots, (**B**) relative expression of *ZmOPR1* in roots, (**C**) relative expression of *ZmAOS1* in shoots, (**D**) relative expression of *ZmSLN1-like* in shoots, (**E**) relative expression of *ZmD9* in shoots of maize. Plants were grown in hydroponic culture under low Mg (0.02 mM) or under normal Mg (0.5 mM) supply and two concentrations of Si (1.5 and 3 mM). Si provided in the second week of plant growth in the hydroponic culture when Mg deficiency was applied. 21-days old plants were harvested 14 days after imposition of Mg deficiency. The white and black bars represent Mg-sufficient and Mg-deficient plants, respectively. Bars indicate means ± SE. Different letters denote significant differences according to LSD test (*p* < 0.05; *n* = 4).

Next, we examined whether Si influences the expression of the genes involved in JA biosynthesis in response to Mg deficiency. Interestingly, the expression of both allene oxide synthase *(ZmAOS1)* and 12-oxophytodienoate 1 (*ZmOPR1)* were significantly enhanced in the roots and shoots of Mg-deficient plants when a higher level of Si was supplied into the solution (Figure 8A–C, Table S1). Moreover, the expression levels of *ZmOPR1* was significantly increased in the roots of Mg-deficient plants with a higher level of Si (Figure 8B). Furthermore, we examined the expression of genes encoding DELLAs in the shoots, which has been reported to be involved in the regulation of environmental signals and hormones particularly activation of JA defense signaling [16,17,18]. The expression of *ZmD9* (dwarf plant 9) and *ZmSLN1-like* (slender 1) were also induced with Si supply in Mg-deficient plants compared to those plant which were solely exposed to Mg deficiency (Figure 8D,E). This induction was even statistically significant in case of *ZmD9* (Figure 8E and Table S1). The same trend was observed for the expression levels of *ZmSLN1-like* under normal Mg and lower concentrations of Si (Figure 8D). These results indicate that Si transcriptionally regulates the expression of DELLA- and JA-related genes under Mg-deficient conditions.

## 3. Discussion

Numerous studies have reported on the role of Si nutrition in ameliorating the adverse effects of biotic and abiotic stresses. In particular, the beneficial effects of Si on mitigating mineral nutrition deficiencies like potassium [9,19], sulfur [9], phosphorus [11,12,13], and also reducing the toxic effects of heavy metals [20,21], have been intensively studied. However, reports elucidating the role of Si under Mg deficiency are lacking. Therefore, in the present study, an attempt was undertaken to study the cross talk between Si and Mg nutrition in hydroponically-grown maize plants. The information obtained on the responses of Mg-deficient plants to Si nutrition is given in the results section, in which the discussion focuses on the regulatory roles of Si supplement in Mg-deficient plants at the physiological, metabolic and hormonal levels.

### 3.1. Silicon Maintained the Growth of Maize Plants Exposed to Mg Deficiency

Mg is involved in many physiological and biochemical processes; thus it is a vital element for plant growth and development [22]. A reduction in plant growth and chlorophyll content has been widely reported under a lack of Mg. For instance, both total dry weight and the chlorophyll content of wheat plants subjected to Mg deficiency decreased by reducing the Mg level [23]. The similar reduction in plant growth and chlorophyll content was also reported in barley plants when the level of Mg reduced [24]. During recent decades, numerous studies have shown that Si maintained or increased plant growth, chlorophyll content and photosynthetic activity in crop plants [20,21]. We have also shown in our previous studies that Si maintained the growth of barley plants subjected to potassium or sulfur deficiencies when combined with osmotic stresses [7,9]. In the present study, and compared to normal Mg supply, the level of chlorophyll, roots and shoots fresh weights decreased under Mg deficiency. Interestingly, Si supply restored the chlorophyll level in Mg-deficient plants to levels comparable to those of Mg-sufficient plants. In addition, the level of chlorophyll significantly increased in Mg-sufficient plants when a higher concentration of Si was provided into nutrient solution (Figure 1C). A similar trend was observed for shoot fresh weight where both concentrations of Si increased the shoot biomass compared to Mg-deficient plants (Figure 1A). The higher shoot biomass by Si might be due to an increase in the water uptake, as Si was shown to increase the leaf water potential in sorghum plants imposed to K deficiency by up-regulating the expression of aquaporin genes and alleviating the decrease of aquaporin activity caused by ROS (reactive oxygen species) [8]. It is worth noting that the root fresh weight significantly decreased under Mg deficiency; however, it seems that Si had a more positive impact on the shoot biomass in Mg-deficient plants. These results are in line with all the aforementioned studies, and indicate that the application of Si maintains the growth of maize plants suffering from Mg deficiency, as supported by higher shoot fresh weight and higher chlorophyll levels.

### 3.2. Silicon Mitigates Mg Deficiency by Primary Metabolites and Polyamine Regulation

As an essential element, Mg plays a vital role in plant metabolism. The fluctuation of plant Mg has a remarkable impact on both transport and utilization of photosynthates, thus significantly impairing carbohydrate partitioning between the source and sink organ [25]. This will lead to a substantial increase in the accumulation of carbohydrates in the leaves of Mg-deficient plants [2], and an increase in the shoot/root ratio. A pioneer report in this field is that of Cakmak et al., 1994 [25], in which the higher accumulation of carbohydrate in beans plants suffering from Mg deficiency was reported. Similarly, in sugar beet plants exposed to Mg deficiency, both starch and Suc accumulated in the rosette leaves [26]. In the present study, as expected, the concentrations of all three soluble carbohydrates Glu, Fru and Suc were significantly increased in the shoots of Mg-deficient plants compared to plants supplied with normal level of Mg (Figure 3E–G). Interestingly, the increase in the levels of all these carbohydrates was significantly and substantially higher in Mg-deficient plants treated with Si. Therefore, one might hypothesize that the observed additional increase in shoot carbohydrates by Si serves as an unfavorable effect of Si under Mg deficiency. Conversely, we believe that the enhanced accumulation of these carbohydrates could rather serve as an osmoticum [27], and also as a signaling mechanism to protect Mg-deficient plants against Mg imbalance. Meanwhile, looking at the concentrations of these carbohydrates in the roots, expect Glu, no reduction was observed in their levels compared to normal Mg plants (Figure 3A–C). This might somehow indicate that the roots were supported by shoot carbohydrates, even under Mg deficiency. This is further supported by the higher expression levels of the genes which are involved in Suc or sugar transport in roots of Mg-deficient plants and in response to Si nutrition (Figure S1). Our finding is perfectly in line with our previously published work on barley plants exposed to K and/or sulfur deficiencies combined with osmotic stress, where Si improved the phloem loading of sugars, mainly Suc, from shoots to roots [7,9]. Altogether, these results indicates that sugars accumulate in Mg-deficient leaves as osmoticum in order to protect the plants from the lack of Mg, and roots might not have suffered from sugar supply when plants were supplied with Si.

The stress tolerance of Mg-deprived maize plants in response to Si nutrition was further investigated with regards to metabolic activity, focusing mainly on those metabolites involved in glycolysis, TCA cycle, polyamines pathways as well as amino acids synthesis. Like soluble sugars, Si also increased the level of hexose-P in the current study with a tandem increase in the levels of citric, isocitric as well as glutamic acids in the shoots of Mg-deficient plants (Figure 5). Interestingly, this increase was associated with enhanced levels of stress amino acids like GABA, Gly and Ser under Mg deficiency when Si was provided in the solution (Figure 4). The role of both Ser and GABA in plant stress tolerance has been reported previously in several studies [28,29]. The modulation of sugars and glycolysis metabolites by Si has also been shown in our previous work on barley plants subjected to combined potassium and osmotic stress where Si increased carbon flux into glycolytic and TCA pathways using higher starch pools [7]. Recently, we have also reported that application of Si to two tomato lines which were different in their response to drought tolerance increased the level of stress amino acids GABA, Gly and Ser; thus, the plant effectively showed greater osmotic stress tolerance [6]. In fact, providing Si for a drought tolerant tomato line increased the levels of amino acids Ser and Gly, which are involved in polyamine synthesis, thereby enhancing drought tolerance in this line. The application of Si to a drought-sensitive tomato line enhanced the levels of GABA and proline, which was associated with an increase in the level of polyamines Put and Spe, leading to drought stress tolerance in this line. Based on these results, we concluded that the involvement of polyamine metabolism was a shared response of both lines to osmotic stress after treatment with Si [6]. Polyamines are known to be involved in stress responses in plants, and particularly in improving drought tolerance [30]. The modulation of polyamine pathways by Si supply was also demonstrated by Yin et al. [16]. These authors have suggested that Si application significantly increased drought stress tolerance in Sorghum plants by increasing the levels of free Put, spermidine and spermidine, and decreasing ethylene levels. In another study in salt-treated Sorghum plants, Si has been shown to enhance salt tolerance by increasing polyamines levels and decreasing ethylene precursor (1-aminocyclopropane-1-carboxylic acid: ACC) [31]. In these studies, Si mainly balanced between polyamines and ethylene levels, thereby enhancing stress tolerance. Interestingly, in the present study, and in agreement with our previous works [6,9], the levels of polyamines Put increased by Si in Mg deficient plants (Figure 6), indicating the modulation of polyamine pathways to cope with the Mg shortage. Therefore, in accordance with our results, we believe that the application of Si under Mg deficiency, increases the sugar pool, which leads to a higher carbon flux into both glycolytic and TCA pathways. This subsequently increases the synthesis of stress amino acids and polyamines to cope effectively with the lack of Mg.

### 3.3. Higher Cytokinin and JA Production by Si Is Associated with Mg Stress Tolerance in Maize Plants

The role of phytohormones has been well documented under nutrient deficiencies [32,33]. Increasing examples are known where there is cross-talk between Si and phytohormones. For example, Si-delayed senescence in Arabidopsis and Sorghum by promoting cytokinin biosynthesis [34]. Also, in another study on Sorghum, Si decreased ACC levels, leading to an increase in polyamines and the retardation of leaf senescence [31]. In our earlier reports, we have also shown the cross talk between Si and ABA in response to sulfur and K deficiencies when combined with osmotic stress [9]. This evidence encouraged us to further investigate the cross talk between Si and phytohormones under Mg deficiency. In the present study, we have found that both IP and IPR level were significantly increased in the shoots of Mg-deficient plants which were treated with higher doses of Si (Figure 7E,F). This is in line with our published work on barley plants exposed to concomitant potassium deficiency and osmotic stress, in which IP levels were significantly increased by Si supply, which positively correlated with the level of chlorophyll and barley senescence marker to delay leaf senescence [7]. The new finding from the present study was a significant increase in shoots JA and JA-ILE concentration by Si, and the latter was also increased in the roots of Mg-deficient plants supplied with Si (Figure 7C,D,G,H). Moreover, the genes involved in JA biosynthesis pathways (*ZmAOS1* and *ZmOPR1*) upregulated by Si supply in response to Mg deficiency in both roots and shoots. This indicates that Si transcriptionally regulated JA biosynthesis pathway for the synthesis of JA in response to Mg deficiency (Figure 8A–C). JA is well known for its role in plant defense responses against insect herbivores and necrotrophic fungi [35]. It is a class of lipid-derived small molecules which plays a key role in the physiological and biochemical responses of plants to environmental conditions [36] against biotic (necrotrophic pathogens and arthropod herbivores), as well as plant responses to abiotic stresses, such as ozone, UV [37] and salinity stress [18]. In the JA biosynthesis pathway, linolenic acid converts to (+)-7-iso-JA via several enzymes. Then, (+)-7-iso-JA rapidly converts to (-)-JA which is more stable and can further convert to many JA conjugates like JA–Ile, which has been suggested to be the endogenous bioactive jasmonate [34]. A pioneering work by Armengaud et al. [38] revealed the cross talk between JA and nutrient deficiencies in Arabidopsis plants; an increase in JA levels in response to potassium deprivation was shown. These authors have also shown the changes in transcript levels of JA-related genes in response to potassium deprivation. The influence of Si treatment on JA concentration has been studied by Kim et al. in rice plants subjected to salinity stress [39]. These authors showed that Si reduced JA levels in salt-treated rice plants in a dose-dependent manner compared to control plants. We believe that the higher JA production with Si supply under Mg deficiency is due to substantial sugar accumulation in the shoots of Mg-deficient plants to protect them from pathogen attack, at least in our experimental conditions. Both Mg and potassium deficiencies increase the concentration of sugars in the leaves [26,37], which are a perfect source for herbivore and fungi attack. Therefore, in the present work, on one hand, Si increased the accumulation of sugars in Mg-deficient plants, using them as compatible solutes to protect themselves from Mg deficiency. On the other hand, we observed a simultaneous increase in JA levels by regulating the genes involved in JA biosynthesis to induce defense responses against pathogens. This is a very interesting observation, since the role of Si on plant pathogens and the underlying mechanism(s) by which Si-mediated the interaction with pathogens are increasingly reported in a number of publications [14,15]. In fact, increasing evidence shows that Si interacts with plant defense signaling components, modulates phytohormones and enhances biochemical resistance, all of which contribute to plant defense responses [14,15]. For instance, in a study on rice, it was shown that Si primed jasmonate-mediated defense responses against rice leaffolder and chewing herbivore, which in turn increased Si accumulation in the leaves of rice, indicating cross-talk between Si and jasmonate in response to insect herbivores [40]. This is in line with our finding in the present study, where Si regulated JA levels in Mg-deficient plants to induce a defense system in response to sugar accumulation. To further confirm whether Si-induced a defense mechanism via JA biosynthesis pathway, we measured the expression level of DELLA proteins. In fact, DELLAs are nuclear proteins that restrain cell proliferation and expansion that induces plant growth [34]. It has been suggested that they integrate plant responses to various hormonal and environmental signals [17]. It was previously shown that how DELLAs bind to ZIM-domain 1 (JAZ1) protein and release MYC2 in order to contribute to JA signaling through JA-responsive genes [41]. DELLAs-JA cross talk has been shown experimentally in a study in Arabidopsis by Shahnejat-Bushehri et al. [35]. These authors have shown the positive interaction between DELLA and JA, and thus, the activation of defense responses against *Pseudomonas syringae*. Interestingly, we showed that the expression of two DELLA genes *ZmD9* and *ZmSLN1-like* were up-regulated in response to Si supply in the shoots of Mg-deficient plants, which can further explain the increase in the levels of JA and JA-ILE to induce plants defenses in response to sugar accumulation. These results indicate that the Mg- deficient plants can profit from Si nutrition to overcome Mg stress by modulating sugar metabolism and further hormonal changes, mainly in JA synthesis.

## 4. Materials and Methods

### 4.1. Plant Materials and Growth Conditions

Seeds of Maize (*Zea mays* cv. ronaldinio) were germinated on vermiculite for 3 days in the dark, followed by a further four days in light conditions. After 7 days, the seedlings were transplanted to a 5.9 L tank in a greenhouse that was set to a 14/10 h day/night cycle at a day/night temperature of 28/25 °C with 40 to 50% relative humidity. Plants were divided into two batches: (i) Mg-sufficient (0.5 mM) plants were grown with a complete solution: Ca(NO_3_)_2_ 1.3 mM, NH_4_NO_3_ 0.7 mM, K_2_SO_4_ 1 mM, MgSO_4_ 0.5 mM, K_2_H_2_PO_4_ 0.2 mM, CaCl_2_ 0.5 mM, H_3_BO_3_ 5 µM, MnSO_4_ 2 µM, ZnSO_4_ 0.5 µM, CuSO_4_ 0.02 µM, (NH_4_)_6_Mo_7_O_24_ 0.01 µM and EDTA, 2NaFe, H_2_O 200 µM. (ii) Mg-deficient plants were grown in the same nutrition solution, but with only 0.02 mM Mg. The nutrient solution was buffered to pH 5.9, and renewed every two days and continuously aerated. In order to avoid osmotic shock, seedlings were grown in 25% strength nutrient solution for the first 2 days, which was raised stepwise to 100% strength within seven days. One week after normal growth, two concentrations of Si (1.5 and 3 mM) were introduced to the culture solution. Monosilicic acid [Si(OH)_4_] was freshly prepared by passing sodium silicate solution through a column filled with cation-exchange resins, according to the method described by Yin et al. [16]. Another set of plants was kept under control conditions without supplying Si. For each treatment, the roots and leaves from 3 independent plants were harvested 21 days after transplanting. The roots and shoots were harvested and immediately frozen in liquid nitrogen and stored at −80 °C until further molecular and biochemical analysis was undertaken. Shoots were ground either in a CryoMill (5 μm, Retsch, Haan, Germany) or manually, maintaining the temperature at −80 °C, and then a part was lyophilised in a Christ Alpha 2–4 LSC system (Christ, Osterode am Harz, Germany). A part of the ground sample was weighed, dried in an oven at 60 °C and further ground with inox beads in an oscillating grinder (5 μm, Mixer Mill MM400; Retsch, Haan, Germany) for elemental analysis. In parallel, root samples were harvested and treated similarly for subsequent analysis.

### 4.2. Chlorophyll Measurement

Leaf chlorophyll content was estimated non-destructively in the last fully expanded leaves (3 plants per treatments) using a portable chlorophyll meter DUALEX- V4 (Force A, Orsay, France).

### 4.3. Determination of Mg and Si

For Si determination, 8 mL of 0.1 M Tiron solution buffered at pH 10.5 was added to 25 mg of DW, which was shaken continuously for 4 h at 65 °C in a shaker incubator (Infors HT, Minitron, Bottmingen, Switzerland). After cooling, 7 mL of H_2_O_2_ (Roth) was added in order to destroy Tiron. The tubes were shaken horizontally in a water bath maintained at 85 °C until the solution turned colorless. The samples were then centrifuged at 4000 rpm for 10 min at 25 °C before analysis. Elements were analyzed by Inductively Coupled Plasma Optical Emission Spectrometry (iCAP 6500 dual OES spectrometer, Thermo Scientific) using an Yttrium solution (1 ppm, Merck, Darmstadt, Germany) as an internal standard.

### 4.4. Phytohormone Determination

Salicylic acid (SA), jasmonic acid (JA), oxo phytodienoic acid (OPDA), jasmonoyl isoleucine (JA-Ile), jasmonoyl phenylalanine (JA-Phe), jasmonoyl valine (JA-Val), abscisic acid (ABA), indole-3-acetic acid (IAA), gibberellin (A1,A2,A4, 7, 8, A19, A24) were all purchased from OlchemIn (Olomouc, Czech Republic). Acide 1-aminocyclopropane-1-carboxylique (ACC) was purchased from Sigma-Aldrich (St Louis, MO, USA). Phaseic acid (PA) and [2H3]-phaseic acid (D-PA) were purchased from (NRC, Ottawa, ON, Canada). ABA, [2H6]-ABA, IAA, and [2H5]-IAA were purchased from OlchemIn (Olomouc, Czech Republic). Phytohormones were analyzed by an UHPLC-MS/MS system. Then, 10 mg FW samples were extracted with 70% methanol, 29% H_2_O, 1% formic acid containing isotope-labelled internal standards, and centrifuged at 12,600 rpm to collect the supernatant. After evaporation (SPE Dry 96, Biotage, Uppsala, Sweden), the extract was re-suspended in 2% formic acid solution and purified using an SPE ABN express plate of 30 mg/mL (Biotage). The phytohormones were eluted with methanol, and samples were evaporated and resuspended in 200 μL of 0.1% formic acid solution before injection into the system. The separation and detection were accomplished using a Nexera X2 UHPLC system (Shimadzu, Japan) coupled to a QTrap 6500+ mass spectrometer (Sciex, Concord, ON, Canada) equipped with an IonDrive turbo V electrospray (ESI) source. Phytohormone separation was carried out by injecting 2 μL into a Kinetex Evo C18 core-shell column (100 × 2.1 mm, 2.6 μm, Phenomenex, Torrance, CA, USA) at a flow rate of 0.7 mL min^−1^, and the column oven was maintained at 40 °C. The mobile phases were composed of solvent A Milli-Q water containing 0.1% formic acid (LCMS grade, Merck, Darmstadt, Germany), and solvent B acetonitrile LCMS grade (Fisher Optima, UK) containing 0.1% formic acid. The analysis was done in scheduled MRM mode in positive.

Isopentenyladenine (IP), isopentenyladenosine (IPA), trans zeatin (TZ), trans zeatin riboside (TZR), cis zeatin (CZ), cis-zeatin riboside (CZR), dihydrozeatin (DHZ), dihydrozeatin riboside (DHZR) were purchased from OlchemIn (Olomouc, Czech Republic). Cytokinins were analyzed by an UHPLC-MS/MS system. Then, 20 mg FW samples were extracted with 70% methanol, 29% H_2_O, 1% formic acid containing isotope-labeled internal standards, and centrifuged at 12,600 rpm to collect the supernatant. After evaporation (SPE Dry 96, Biotage, Uppsala, Sweden), the extract was re-suspended in 2% formic acid solution and purified using an SPE CX express plate of 30 mg/mL (Biotage, Uppsala, Sweden). The cytokinins were eluted with 5% ammonium hydroxide methanolic solution and samples were evaporated and re-suspended in 100 μL of 0.1% formic acid solution before injection into the system. Separation and detection conditions were determined using the same parameters described above.

### 4.5. Determination of Primary Metabolites and Polyamines

Soluble sugar determination was undertaken according to the method described by Kim et al., 2013 [18]. Next, 10 mg frozen shoot material were homogenized in liquid nitrogen, dissolved in 0.75 mL of 80% (*v*/*v*) ethanol and incubated at 80 °C for 60 min. Crude extracts were centrifuged at 14,000 rpm for 10 min at 4 °C, and the upper phase was concentrated in a SpeedVac concentrator (Thermo Scientific, Waltham, MA, USA) at 45 °C for 180 min. The pellet was re-suspended in 0.75 mL deionized water and incubated at 80 °C for 60 min. After centrifugation, the second supernatant was added to the first and concentrated. Hexokinase (HK), phosphoglucoisomerase (PGI) and b-fructosidase were added successively to measure Glc, Fru and Suc, as described in Kim et al., 2013 [42].

For amino acid determination, 10 mg lyophilized dry matter was extracted with a solution containing of 400 µL of MeOH and 0.625 nmol/µL Norvaline, which was used as the internal standard (Sigma Aldrich, St. Louis, MO, USA). Extraction was stirred for 15 min, and was then re-suspended with 200 µL of chloroform and 400 µL of ddH_2_O. After centrifugation (12,000 rpm, 10 °C, 5 min), the supernatant was recovered, evaporated and dissolved in 100 µL of ddH_2_O. Derivatization was performed using an Ultra Derivatization Kit AccQ tag, following the manufacturer’s protocol (Waters Corp, Milford, MA, USA). The amino acid profile was determined by using UPLC/PDA H-Class system with BEH C18 100 ×2.1 mm column.

Metabolite analysis was performed as described previously by Ali et al., 2018 [8]. Metabolite extraction was conducted using 30 mg of frozen grinded fresh leaves and roots, which were weighed in a 2 mL Eppendorf tubes, then 500 µL of cold water/methanol 70:30 *v*/*v* (−20 °C) containing 0.1% of perchloric acid (*v*/*v*) solvent were added. Samples were shaken with vortex for 20 min. Then, they were centrifuged using an Eppendorf Centrifuge 5427 R (Eppendorf, Hamburg, Germany) for 15 min 12,700 rpm at 4 °C. Supernatants were collected and introduced in a new 2 mL Eppendorf tubes. A second extraction was performed adding 500 µL of water + 0.1% perchloric acid (*v*/*v*) to leaves and roots, shaken for 5 min with vortex, and centrifuged for 15 min with 12,700 rpm on 4 °C. Supernatants were mixed and centrifuged for 10 min in order to eliminate suspended particles. Finally, supernatants were diluted 3 times with water + 0.1% Formic acid (*v*/*v*) and introduced in 2 mL LC-MS vials. Metabolite analysis was achieved using an ultra-high performance liquid chromatography (UPLC) Acquity H-Class system (Waters Corp, Milford, MA, USA), and high-resolution detection was performed by using a Xevo G2-S QToF mass spectrometer (Waters Corp, Milford, MA, USA) equipped with an electrospray ionization (ESI) source. A Phenomenex Luna^®^ Omega PS C18 (100 × 2.1 mm, 1.6 µm) column (Torrance, CA, USA) was used to profile metabolites such as organic acids. The mobile phase, comprising water containing 0.5% formic acid (A) and methanol: water (70:30 *v*/*v*) containing 0.5% formic acid (B), was applied with the optimized gradient elution as follows: 100% A at 0–1 min, 100–20% A at 1–4 min, 20–0% A at 4–6.5 min, 0% A at 6.5–7.5 min, 0–100% A at 7.5–7.9 min, 100% A at 7.9–10 min. The flow rate was kept at 0.3 mL/min, column temperature was maintained at 35 °C. The injection volume for both columns was 10 µL and samples were maintained at 10 °C. The ESI source was used in negative ionization, source voltage was set to 2.5 kV and cone voltage was 30 V, whilst source temperature was maintained at 130 °C with a cone gas flow of 20 L/h. The desolvation temperature was at 550 °C, with a desolvation gas flow of 900 L/h. Leucine-Enkephalin was used as lockmass reference, (ion at *m*/*z* 556.2771 in positive mode), which was introduced by a lockspray at 10 μL min^−1^ for real-time data calibration. The MSE data were acquired in centroid mode using a scan range 50–800 Da, scan time 0.1 s, resolution was set at 20000 full width half maximum (FWHM), and a collision energy ramp 40–80 V.

Polyamine extraction was achieved using 20 mg of frozen grounded leaves that were weighed in a 2 mL eppendorf tube (Eppendorf, Hamburg, Germany). Extraction was carried out by adding 1 mL of a solution of 70% H_2_O/29% MeOH/1.0% formic acid (*v*/*v*/*v*) at −20 °C. Next, the tubes were stirred at room temperature for 30 min, and then centrifuged at 4 °C (16,000 rpm), and the supernatant was transferred into new Eppendorf tubes. The supernatant was transferred to a LC/MS vial for analysis. Polyamines were analyzed by an UHPLC–MS/MS system. The separation and detection were achieved using a Nexera X_2_ UHPLC system (Shimadzu, Kyoto, Japan) coupled to a QTrap 6500+ mass spectrometer (Sciex, Concord, ON, Canada) equipped with an IonDrive^TM^ turbo V electrospray (ESI) source.

### 4.6. RNA Extraction And Gene Expression Analysis

The roots and shoots sample (100 mg) of maize plants were grounded to a fine powder in the presence of liquid nitrogen and total RNA was extracted using a Nucleospin^®^ 8 RNA kit following the manufacturer’s protocol (Macherey-Nagel, Düren, Germany). The quality and yield of all RNA samples were analyzed and checked in a 4200 Tapestation (Agilent Technologies, Santa Clara, CA, USA), followed by DNase treatment and cDNA synthesis from 1 μg RNA using iScript^TM^ gDNA clear cDNA synthesis kit (Bio-Rad, Hercules, CA, USA). Quantitative RT-PCR (qPCR) analysis was performed in a total volume of 10 μL using Universal SYBR Green Supermix (Bio-Rad, Hercules, CA, USA) in Real-Time PCR Detection System (Bio-Rad, Hercules, CA, USA). The qPCR reactions were performed in technical triplicates using independent cDNA reactions for each biological replicate and 300 nM of gene-specific primer pairs. Specific primers for all candidate genes were designed using Primer3 software (version 0.4.0) and are listed in Table S5. The thermal cycler protocol was 98 °C for 3 min, 40 cycles of 98 °C for 15 s, 60 °C for 30 s, 72 °C for 15 s and a final 5-min extension at 72 °C. The expression of all candidate genes were normalized against four maize reference genes, namely, *GAPDH*, *CYCLOPHILIN*, *EIF4a* and *β-tubulin*. All qPCR expression data were acquired and analyzed using CFX Maestro Software Version 1.0 (Bio-Rad, Hercules, CA, USA).

### 4.7. Statistical Analysis

For individual treatment, hydroponic experimentations on *Zea mays* were conducted under control and osmotic stress conditions, with 4 independent biological replicates consisting of 3 plants each. Data are represented as mean ± standard error (SE) for *n* = 4. The LSD test (R software) was employed to analyze all the data and marked by different letters when significantly different (*p* < 0.05).

## 5. Conclusions

**Figure 9:**
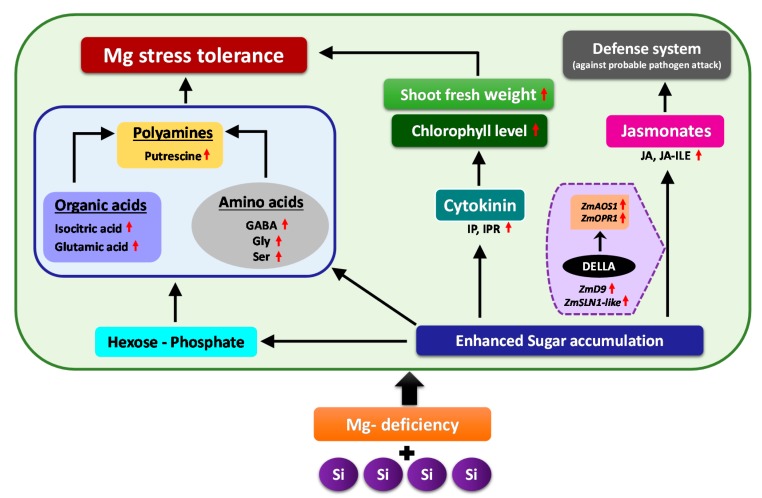
Schematic model illustrating the response of Mg-deficient maize plants to Si supply. Application of Si to the Mg-deficient maize plants enabled the plants to cope better with Mg deficiency by regulating primary metabolites and inducing phytohormonal changes. Si induced multiple responses under Mg deficiency, whereof the first response was enhanced accumulation of sugars in the shoots as an osmoticum that was accompanied by a simultaneous increase in hexose-P levels, organic acids, stress amino acids as well as polyamines (Putrescine), which sufficiently provided the carbon flow to glycolytic pathway. The second response was related to the phytohormonal regulation, due to which specific changes were observed in the level of active cytokinins IP and IPR as well as JA and its derivative JA-ILE. The increased concentration of cytokinin maintained the plant growth under Mg-deficient condition as indicated by higher shoot fresh weights and augmented chlorophyll level, whereas the increased level of jasmonates switched on the plant defense system (against probable pathogen attack) in response to enhanced sugar accumulation which was correlated with higher expression of DELLAs that might have further induced the JA related genes. Altogether, these responses contributed to Mg stress tolerance in the maize plants (Red arrows = increase/upregulation).

In this study, for the first time, we report the cross talk between Mg and Si nutrition. We showed that Si supply to maize plants exposed to Mg deficiency regulated plant primary metabolites and modulated the phytohormones levels to cope with Mg deficiency, thus maintaining plants growth and development. The first response of Mg-deficient plants to Si nutrition was the accumulation of soluble sugars as compatible solutes, as well as the increase in hexose-P. This increase provided carbon flow toward glycolytic pathway, and consequently, increased the level of organic acids, synthesis of stress amino acids GABA, Gly and Ser as well as polyamines Put which all contributed to Mg stress tolerance. The second response to Si nutrition under Mg deficiency was associated with phytohormonal changes—mainly an increase in the levels of cytokinin, JA and its derivate JA-ILE. Higher cytokinin maintained the plants’ growth and development under Mg deficiency, as indicated by higher shoot fresh weight. Moreover, the increase in the level of JA and JA-ILE switched on the plant defense system (against probable pathogens attack) in response to substantial sugar accumulation in the shoots, which was associated with higher expression of the DELLAs which induced JA related genes. Overall, our findings revealed that the application of Si increases the tolerance of maize plants subjected to Mg deficiency by regulating primary metabolite and the hormonal balance (Figure 9).

## Supplementary Materials

Supplementary materials can be found at https://www.mdpi.com/1422-0067/20/4/969/s1. Table S1. Two-way ANOVA analysis for Mg and Si interaction. Table S2. Metabolite concentration in roots. Table S3. Metabolite concentrations in shoots. Table S4. Hormone concentrations in roots and shoots. Table S5. List of the primers. Figure S1. Expression level of the genes involved in sugars transporter.

## Abbreviations

JA	Jasmonic acid
JA-ILE	L-isoleucine
AGAB	Gammaaminobutyric acid
LD	linear dichroism
Glu	Glucose
Fru	Fructose
Suc	Sucrose
Gly	Glycine
Ser	Serine
TCA	Tricarboxylic acid cycle
Put	Puterscine
Spe	Spermidine
Ip	Iso-pentenyladenine
IPR	Iso-pentenyladenine riboside
ABA	Abscisic acid
ACC	1-aminocyclopropane-1-carboxylic acid
SA	Salicylic acid
GA	Gibberellin acid
