# Supplementary material for: The Ameliorative Effect of Silicon on Maize Plants Grown in Mg-Deficient Conditions
Source: Int J Mol Sci. 2019 Feb 22;20(4):969. doi: 10.3390/ijms20040969 (PMC6412671; doi:10.3390/ijms20040969)
Supplement: Supplementary file 1 [file ijms-20-00969-s001.zip › ijms-441644-supp materials_corrections.pdf]

International Journal of
Molecular Sciences

MDPI

Table S2. Influence of Si supply on metabolite concentrations in roots of maize plants subjected to Mg deficiency.

		+Mg			-Mg		
	**Metabolite**	**-Si**	**+Si 1.5**	**+Si 3**	**-Si**	**+Si 1.5**	**+Si 3**
**Amino acids**	Asn	2.79 a	2.67 a	2.67 a	2.67 a	2.37 a	2.94 a
	His	0.25 a	0.23 b	0.23 bc	0.21 c	0.21 c	0.21 c
	Ser	1.30 a	1.23 a	1.14 a	1.29 a	1.19 a	1.34 a
	Gln	3.56 a	3.48 a	3.06 ab	2.91 ab	2.66 b	3.02 ab
	Gly	0.28 ab	0.28 ab	0.25 b	0.34 a	0.31 ab	0.34 ab
	Arg	2.41 a	2.24 ab	1.80 bc	1.51 c	1.57 c	1.62 c
	Asp	0.19 a	0.15 bc	0.16 b	0.13 c	0.14 bc	0.15 bc
	Thr	0.63 a	0.57 ab	0.57 ab	0.49 bc	0.44 c	0.51 bc
	Ala	1.25 a	1.13 a	1.05 a	1.31 a	1.26 a	1.34 a
	GABA	0.17 b	0.17 b	0.22 ab	0.26 a	0.26 a	0.28 a
	Pro	0.11 abc	0.09 c	0.09 bc	0.10 abc	0.12 ab	0.12 a
	Lys	0.14 a	0.14 ab	0.14 ab	0.12 b	0.13 ab	0.14 a
	Tyr	0.64 a	0.72 a	0.65 a	0.68 a	0.66 a	0.68 a
	Val	0.34 a	0.34 ab	0.32 abc	0.29 bc	0.29 c	0.31 abc
	Ile	0.17 a	0.17 a	0.18 a	0.16 a	0.17 a	0.17 a
	Leu	0.23 ab	0.23 ab	0.25 a	0.21 b	0.22 b	0.20 b
	Phe	0.18 a	0.20 a	0.18 a	0.17 a	0.16 a	0.16 a
**TCA cycle**	Fumarate	22.4 a	14.3 b	17.7 b	14.1 b	13.9 b	15.9 b
	Malate	16.3 a	9.05 c	12.9 b	10.1 bc	11.1 bc	13.0 b
	Citrate	6.75 a	5.19 ab	5.84 a	3.35 b	3.52 b	3.61 b
	Isocitrate	3.63 a	2.79 ab	3.08 ab	2.53 ab	1.59 b	3.12 ab
**Glycolysis**	Hexose-P	41.4 ab	41.5 ab	45.7 a	41.2 ab	37.5 b	43.5 ab
	PEP	0.94 b	1.20 a	0.92 b	1.05 ab	0.97 b	0.97 b
	Glutamate	94.3 a	91.1 ab	89.1 ab	76.4 bc	69.1 c	75.1 bc
	Aspartate	265.7 ab	275.7 a	213.2 bc	211.0 c	198.2 c	223.5 abc
	Acide Gluconique	7.82 c	7.74 c	7.83 c	9.11 bc	11.3 ab	12.9 a

Concentrations of amino acids and primary metabolites were measured in roots of maize. Plants were grown in hydroponic culture under low Mg (0.02 mM) or normal Mg (0.5 mM) supply and two concentrations of Si (1.5 and 3 mM). Si provided in the second week of plant growth in the hydroponic culture when Mg deficiency was applied. 21-days old plants were harvested 14 days after imposition of Mg deficiency. Bars indicate means ± SE. Different letters denote significant differences according to LSD test ($p < 0.05$; $n = 4$). The concentration of metabolites calculated based on mg/g FW. Asn, asparagine; His, histidine; Ser, serine; Gln, glutamine; Gly, glycine; Arg, arginine; Asp, aspartate; Thr, threonine; Ala, alanine; GABA, gammaaminobutyricacid; Pro, proline; Lys, lysine; Tyr, tyrosine; Val, valine; Ile, isoleucine; Leu, Leucine; Phe, phenylalanine; Hexose-6-P, hexose-6-phosphate; PEP, phosphoenolpyruvate.

Table S3. Influence of Si supply on metabolite concentrations in shoots of maize plants subjected to Mg deficiency.

		+Mg			-Mg		
	**Metabolite**	**-Si**	**+Si 1.5**	**+Si 3**	**-Si**	**+Si 1.5**	**+Si 3**
**Amino acids**	Asn	0.89 ab	0.74 b	0.96 ab	1.03 ab	0.97 ab	1.09 a
	His	0.10 c	0.10 c	0.10 c	0.16 b	0.19 a	0.18 ab
	Ser	1.85 c	1.73 c	1.84 c	2.76 b	3.10 ab	3.43 a
	Gln	0.82 b	0.88 b	0.94 ab	1.07 ab	1.04 ab	1.16 a
	Gly	1.00 c	0.65 c	0.79 c	2.04 b	3.59 a	3.15 a
	Arg	2.01 a	1.95 a	1.99 a	1.46 b	1.19 b	1.30 b
	Asp	0.09 ab	0.10 a	0.10 a	0.09 ab	0.07 b	0.08 ab
	Thr	0.48 bc	0.44 c	0.47 c	0.55 ab	0.58 a	0.61 a
	Ala	2.19 ab	1.85 b	1.96 b	2.20 ab	2.60 a	2.61 a
	GABA	0.07 bc	0.05 d	0.07 c	0.07 bc	0.09 ab	0.10 a
	Pro	0.06 b	0.05 b	0.05 b	0.13 a	0.16 a	0.14 a
	Lys	0.14 a	0.14 a	0.15 a	0.17 a	0.18 a	0.18 a
	Tyr	0.18 c	0.25 c	0.18 c	1.14 b	1.51 a	1.25 ab
	Val	0.12 b	0.11 b	0.11 b	0.34 a	0.44 a	0.40 a
	Ile	0.07 c	0.06 c	0.06 c	0.27 b	0.38 a	0.34 ab
	Leu	0.06 b	0.06 b	0.06 b	0.28 a	0.35 a	0.33 a
	Phe	0.04 c	0.04 c	0.04 c	0.11 b	0.16 a	0.15 ab
**TCA cycle**	Fumarate	82.9 a	77.6 ab	74.0 ab	66.2 b	52.3 c	68.7 ab
	Malate	62.1 a	42.9 bc	45.6 b	37.7 bc	32.3 c	44.1 b
	Citrate	14.0 ab	15.4 a	13.7 ab	9.66 c	11.7 bc	11.9 bc
	Isocitrate	4.94 c	5.47 c	4.60 c	24.4 b	42.8 a	44.5 a
**Glycolysis**	Hexose-P	32.7 b	33.8 b	32.5 b	40.1 b	51.6 a	58.5 a
	PEP	6.49 a	6.68 a	5.83 a	3.20 b	2.91 b	3.03 b
	Glutamate	81.8 a	75.4 a	73.4 a	57.4 b	56.2 b	74.3 a
	Aspartate	197.6 a	182.1 a	179.8 a	125.9 b	102.6 b	133.8 b
	Acide Gluconique	4.99 c	5.08 c	4.66 c	5.94 bc	7.86 a	7.27 ab

Concentrations of amino acids and primary metabolites were measured in shoots of maize. Plants were grown in hydroponic culture under low Mg (0.02 mM) or normal Mg (0.5 mM) supply and two concentrations of Si (1.5 and 3 mM). Si provided in the second week of plant growth in the hydroponic culture when Mg deficiency was applied. 21-days old plants were harvested 14 days after imposition of Mg deficiency. Bars indicate means ± SE. Different letters denote significant differences according to LSD test ($p < 0.05$; $n = 4$). The concentration of metabolites calculated based on mg/g FW. Asn, asparagine; His, histidine; Ser, serine; Gln, glutamine; Gly, glycine; Arg, arginine; Asp, aspartate; Thr, threonine; Ala, alanine; GABA, gammaaminobutyricacid; Pro, proline; Lys, lysine; Tyr, tyrosine; Val, valine; Ile, isoleucine; Leu, Leucine; Phe, phenylalanine; Hexose-6-P, hexose-6-phosphate; PEP, phosphoenolpyruvate.

Table S4. Influence of Si supply on hormone concentrations in roots and shoots of maize plants subjected to Mg deficiency.

	Hormones	+Mg			-Mg		
		-Si	+Si 1.5	+Si 3	-Si	+Si 1.5	+Si 3
Roots	ABA	4.35 a	3.26 a	3.49 a	4.31 a	3.41 a	3.67 a
	SA	12.6 a	5.80 c	5.27 c	7.17 b	6.01 bc	12.9 a
	GA19	8.84 a	7.97 a	7.00 a	7.25 a	4.27 a	7.30 a
	ACC	65.9 a	69.3 a	74.7 a	69.1 a	79.2 a	67.0 a
	IAA	2.84 a	2.67 a	2.43 ab	2.26 ab	2.19 ab	2.05 b
Shoots	ABA	9.93 a	10.4 a	9.55 a	9.30 a	9.42 a	9.53 a
	SA	10.0 a	8.72 a	11.3 a	9.86 a	9.12 a	9.37 a
	GA19	26.3 a	21.4 ab	25.5 a	21.2 ab	18.6 b	21.1 ab
	ACC	42.1 a	38.3 ab	39.8 a	31.6 c	32.7 bc	31.7 c
	IAA	3.84 a	4.16 a	3.91 a	4.81 a	4.31 a	4.31 a

Concentrations of hormones were measured in roots and shoots of maize. Plants were grown in hydroponic culture under low Mg (0.02 mM) or normal Mg (0.5 mM) supply and two concentrations of Si (1.5 and 3 mM). Si provided in the second week of plant growth in the hydroponic culture when Mg deficiency was applied. 21-days old plants were harvested 14 days after imposition of Mg deficiency. Bars indicate means ± SE. Different letters denote significant differences according to LSD test ($p < 0.05$; $n = 4$). The concentration of hormones calculated based on pg/mg FW. ABA, abscisic acid; SA, salicylic acid; GA19, gibberellic acid 19; ACC, 1-Aminocyclopropane-1-carboxylic acid; IAA, Indole-3-acetic acid.

Table 5. List of primers used in this study.

Gene	Accession No.	Forward Primer	Reverse Primer	Amplicon Size (bp)
ZmSLN1-like	NM_001322273	5'-ATGCTGGTTGGGCTCTTCT-3'	5'-CTGCTCCTGCTGTCACTCT-3'	143
ZmD9	DQ903073	5'-TTCTACGAGTCCTGCCCC-3'	5'-CCCTGCTTGATGCCGAAG-3'	116
ZmAOS1	XM_008646248	5'-GCTGGTCAAGAAGGACTACGA-3'	5'-AAGCACATGGCGAAGAGGA-3'	132
ZmOPR1	NM_001112429	5'-ACCGCTCCACCTTCTACAC-3'	5'-CTGACTCCTCATTCTTGCCATC-3'	90
ZmSUT1	NM_001111370	5'-TTTCTGGTGGCTGTGGTGT-3'	5'-TTTGTGGGGAGGTTCTGGTT-3'	98
ZmSWEET13a	NM_001155615	5'-GGCGTTTGCTTTCGGTCT-3'	5'-CTTGCTCTTGTAGATGCGGT-3'	91
ZmSWEET13b	NM_001148182	5'-ATCCAGACGAAGAGCGTAGA-3'	5'-CCGTAGAGGAACCAGACGA-3'	83
ZmSWEET13c	NM_001147634	5'-ACCAAGAAGGGCAGGATGTT-3'	5'-AGAAGGGTGAGGAGAAGGATG-3'	86
ZmEIF4A	AF007580	5′-GACAAGATGAGGAGCAGGGA-3′	5′-CAATACCACGAGCAAGCAGG-3′	142
ZmGAPDH	NM_001111943	5′-ACAGCGACATCACACTCAAGG-3′	5′-GACTCCACGACATACTCAGCG-3′	127
ZmCYP	M55021	5′-ACGGCTCCCAGTTCTTCATC-3′	5′-CAGCGACCTTGACCACCTT-3′	156
Zmβ-tub	NM_001111987	5′-GAGGTGGACGAGCAGATGA-3′	5′-CACACGCTGGACTTGACATT-3′	89

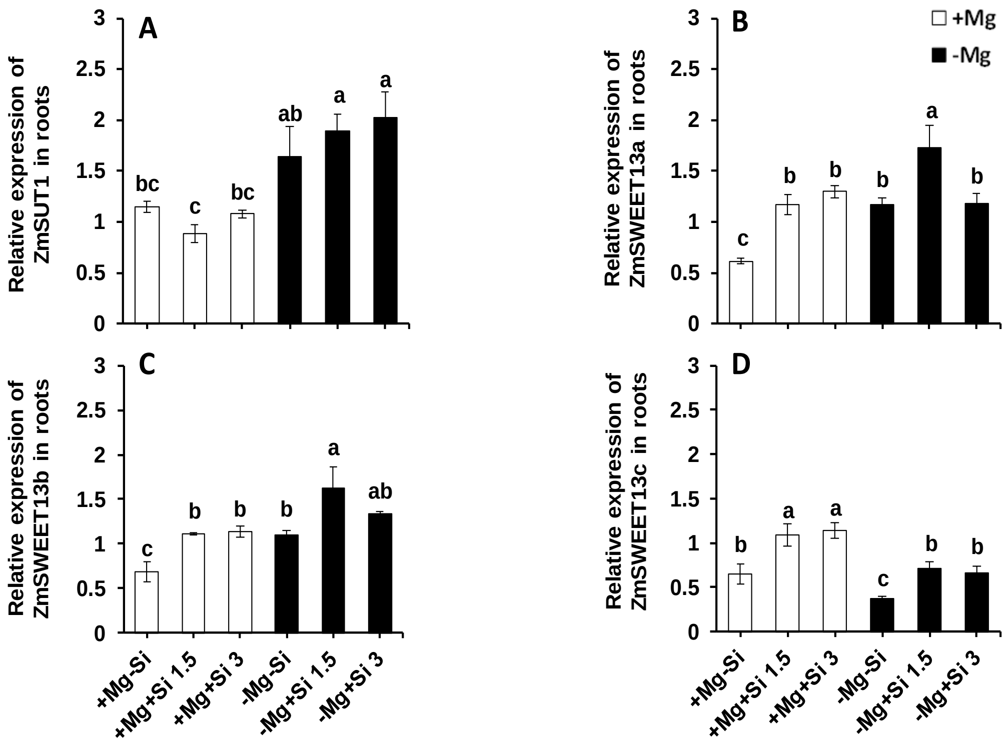

Figure S1. Influence of Si supply on the expression of the genes involved in Suc and sugar transporter of maize plants subjected to Mg deficiency. (A) Relative expression of ZmSUT1 in roots, (B) relative expression of ZmSWEET13a in roots, (C) relative expression of ZmSWEET13b in roots and (D) relative expression of ZmSWEET13c in roots of maize. Plants were grown in hydroponic culture under low Mg (0.02 mM) or normal Mg (0.5 mM) supply and two concentrations of Si (1.5 and 3 mM). Si provided in the second week of plant growth in the hydroponic culture when Mg deficiency was applied. 21-days old plants were harvested 14 days after imposition of Mg deficiency. The white and black bars represent Mg-sufficient and Mg-deficient plants, respectively. Bars indicate means ± SE. Different letters denote significant differences according to LSD test ($p < 0.05$; $n = 4$).
